# Study on the differences of gut microbiota composition between phlegm-dampness syndrome and qi-yin deficiency syndrome in patients with metabolic syndrome

**DOI:** 10.3389/fendo.2022.1063579

**Published:** 2022-11-09

**Authors:** Haonan Shang, Lu Zhang, Tiegang Xiao, Li Zhang, Jun Ruan, Qiang Zhang, Kaili Liu, Zhonghai Yu, Yueqiong Ni, Bing Wang

**Affiliations:** ^1^ Department of Traditional Chinese Medicine, Shanghai Sixth People’s Hospital Affiliated to Shanghai Jiao Tong University School of Medicine, Shanghai, China; ^2^ Systems Biology & Bioinformatics Unit, Leibniz Institute for Natural Product Research and Infection Biology - Hans Knöll Institute, Jena, Germany; ^3^ Shanghai municipal Hospital of Traditional Chinese Medicine, Shanghai University of Traditional Chinese Medicine, Shanghai, China

**Keywords:** metabolic syndrome, traditional Chinese medicine, gut microbiota, phlegm-dampness syndrome, qi-yin deficiency

## Abstract

**Background:**

Metabolic syndrome (MS) is a group of complex medical conditions that can lead to serious cardiovascular and cerebrovascular diseases. According to the theory of traditional Chinese medicine (TCM), MS can be divided into two main subtypes termed ‘phlegm-dampness syndrome’ (TSZE) and ‘qi-yin deficiency syndrome’ (QYLX). At present, the research into intestinal microbiota of different TCM syndromes of MS and its association with clinical manifestation is lacking.

**Materials and methods:**

Using 16S rRNA sequencing, we performed a cross-sectional analysis of human gut microbiota between two different TCM syndromes (QYLX and TSZE, n=60) of MS, and their differences with healthy participants (n=30).

**Results:**

We found that the QYLX and TSZE groups differ from the healthy control group in the overall gut microbiota composition, and some specific microbial taxa and functional pathways. Moreover, significantly differentially abundant taxa and distinct BMI-correlated taxa were observed between QYLX and TSZE groups, suggesting the potential contribution of gut microbiota to the distinction between the two TCM syndromes. The predicted functional profiles also showed considerable differences, especially pathways related to amino acid metabolism and lipopolysaccharide synthesis.

**Conclusion:**

Our study highlights the gut microbiota’s contribution to the differentiation between two TCM syndromes of MS and may provide the rationale for adopting different microbiota-directed treatment strategies for different TCM syndromes of MS in the future.

## Introduction

Metabolic syndrome (MS) is a clustering of risk factors, such as central obesity, insulin resistance, dyslipidaemia and hypertension that together culminate in the increased risk of type II diabetes mellitus and cardiovascular disease (1). Available evidences show that in most countries, 20% to 30% of the adult population can be described as suffering from metabolic syndromes, and the prevalence is even higher in some countries (2). Unlike modern medicine, the understanding of MS in TCM involves viscera, qi and blood, body fluid, yin and yang, and is related to many pathological products such as phlegm, dampness, heat and blood stasis (3, 4). According to TCM, the yin and yang in harmonious balance indicate healthiness, whereas imbalances towards either side indicate unhealthiness, which may result in diseases (5). The syndrome of TCM is the whole reflected state of pathology and physiology in the background of a disease (6). It is generally considered that metabolic syndrome has 5 TCM syndromes, among which the most common is qi-yin deficiency syndrome, followed by phlegm-dampness syndrome, yin deficiency heat excess syndrome, phlegm-stasis interaccumulation syndrome, and yin and yang deficiency syndrome (7, 8).

The collection of bacteria, archaea, viruses and eukaryote colonizing the gastrointestinal tract is termed ‘gut microbiota’. It constitutes a huge and complex microecosystem that plays an important role in the host (9). In the long-term co-evolution of gut microbiota and human beings, there is always a dynamic balance between different microbial strains, hosts, and external environment (10), reaching finally a relatively stable ‘internal environment’. This dynamic balance involves various ecological relationships such as mutualism and competition, and is similar to the theory of yin and yang balance in TCM, which includes the interdependence between yin and yang, the opposition and restriction of yin and yang, and the absence of single yin or yang. During health, the intestinal microbiota provides many benefits to the host and is generally resistant to colonization by new species; however, disruption of this complex community can lead to pathogen invasion, inflammation, and disease (11). Because the MS is a cluster of different conditions rather than a single symptom, its pathogenesis and clinical manifestations are complex. As such, studying the involvement of gut microbiota in MS, especially in relation to TCM, could increase our understanding of this complex systemic disease.

Previous studies have found differences in the TCM syndromes of some diseases compared with controls (12), as well as differences in gut microbiota between TCM syndromes, such as ulcerative colitis and colorectal cancer (13, 14), but there has been no study on intestinal microbiota of different syndrome types of MS. Therefore, we selected the two most common TCM syndromes of MS, phlegm-dampness syndrome and qi-yin deficiency syndrome, in order to study whether there exist differences in gut microbiota among MS patients with different TCM syndromes. We collected stool samples from 60 MS patients together with 30 healthy control participants and used 16S rRNA sequencing technology to investigate the overall microbiota composition and specific microbial abundances. We found significant differences in the gut microbiota between the TCM syndrome of MS and healthy participants, as well as between patients with the two different TCM syndromes, thus providing the rationale for adopting different microbiota-directed treatment strategies for different TCM syndromes. This study was approved by the Medical Ethics Committee of Shanghai Sixth People’s Hospital (project acceptance number: SH6THHOSP-NDGZ-2019-035).

## Materials and methods

### Diagnosis of metabolic syndrome

Metabolic syndrome was diagnosed using the 2013 Chinese Diabetes Society criteria (15) (Diabetes Branch of Chinese Medical Association), by the presence of three or more following conditions: (1) central obesity: waist circumference (WC) (Chinese) for male ≥ 90 cm or female ≥ 85 cm; (2) hyperglycemia: fasting plasma glucose ≥ 6.1 mmol/L and/or 2 h plasma glucose ≥ 7.8 mmol/L, or previously diagnosed type II diabetes and receiving treatment; (3) hypertension: systolic blood pressure/diastolic blood pressure ≥ 130/90 mmHg, or previously diagnosed hypertension and receiving treatment; (4) fasting plasma triglyceride ≥ 1.7 mmol/L (150mg/dL); (5) fasting plasma high-density lipoprotein cholesterol (HDL) < 1.04 mmol/L.

### Defining TCM syndromes of MS

By referring to Guiding principles for clinical research of new syndromic TCM drugs (16) from National Medical Products Administration, combined with clinical observation and literature reports, we selected two common syndromes of deficiency and/or excess: (1) Qi-yin deficiency syndrome (QYLX), with main symptoms being shortness of breath, fatigued spirit and weakness, chest discomfort and vague pain sometime, and minor symptoms being dizziness and palpitation, dysphoria in chest palms-soles, spontaneous sweating or night sweating, thirst with desire for drinks, short voidings of scant urine, dry and hard stool, red tongue with little coating or teeth-printed tongue, vacuous pulse or irregularly pulse; (2) Phlegm-dampness syndrome (TSZE), with main symptoms being heaviness of head, sense of suppression in the chest, numbness and heaviness in limbs, and minor symptoms including obesity, palpitation, tastelessness and poor appetite, nausea and salivation, sleepiness, facial distortion, tongue with white greasy or slippery fur, smooth pulse. Patients with MS were diagnosed with either QYLX or TSZE if two main symptoms or one main symptom plus two concurrent minor symptoms are present.

### Patient recruitment

From March 2018 to May 2019, 140 patients with MS were collected from the outpatient and inpatient departments of Shanghai Sixth People’s Hospital Affiliated to Shanghai Jiao Tong University School of Medicine. All patients filled in the questionnaire for clinical information on TCM syndromes of metabolic syndrome and 60 patients who met all inclusion and exclusion criteria were enrolled in this clinical study. In addition, healthy control participants were recruited from participants undergoing health examination in Shanghai Sixth People’s Hospital Affiliated to Shanghai Jiao Tong University School of Medicine (all healthy participants in the control group had no MS after medical diagnosis), including 16 males and 14 females. This study was approved by the Medical Ethics Committee of Shanghai Sixth People’s Hospital (project acceptance number: SH6THHOSP-NDGZ-2019-035).

Detailed inclusion criteria were: (1) Men and women aged 18-65 (inclusive); (2) Non-pregnant and non-lactating; (3) Conformed to the diagnostic criteria for MS established in the above-mentioned western medicine standards; (4) Met the diagnostic criteria of qi-yin deficiency syndrome or phlegm-dampness syndrome in the TCM syndrome types of MS formulated in the above TCM standards; (5) No history of using microecological agents (probiotics or prebiotics) or antibiotics within 2 weeks before the sampling; (6) Willing to follow the sampling method of this study and to provide qualified samples; (7) Submitted the signed informed consent before the experiment and being cooperative in the whole process of the experiment.

Participant who has any one or more than one of the following conditions should be excluded from this study: (1) Has taken microecological agents (probiotics or prebiotics) or antibiotics within 2 weeks before sampling; (2) Infected with infectious diseases, or severe trauma or operation; (3) Contaminated specimens during or after sampling; (4) Pregnant or lactating women.

### Fecal sample collection

The fresh feces naturally discharged by all candidates were collected (more than 2g), quickly put into sterile sample tubes and then transported to the Spleen and Stomach Disease Laboratory of Longhua Hospital within 1 hour with ice bath. Samples were unpacked under sterile conditions and stored in -80°C ultra-low temperature refrigerator after cooling with liquid nitrogen.

### DNA extraction, library preparation and Illumina MiSeq PE250 sequencing

Microbial DNA was extracted from the samples using the QIAamp fast DNA stool Mini Kit (51604, Qiagen, Germany) according to the manufacturer’s protocols. The V3-V4 regions of bacterial 16S ribosomal RNA genes were amplified with PCR (95°C for 3 minutes, followed by 30 cycles at 98°C for 20s, 58°C for 15s, 72°C for 20s and the last extension step at 72°C for 5 minutes) using primers 341F 5’-CCTACGGGRSGCAGCAG)-3’ and 806R 5’-GGACTACVVGGGTATCTAATC-3’. PCR reactions were carried out in 30 μL of mixture containing 15 μL of 2 × Kapa Library Amplification ReadyMix, 1 μL of each primer (10 μM), 50ng template DNA and ddH2O.

Amplicons were extracted from 2% agarose gels and purified using the AxyPrep DNA Gel Extraction Kit (Axygen Biosciences, Union City, CA, U.S.) according to the manufacturer’s instructions and quantified using Qubit^®^2.0 (Invitrogen, U.S.). After the preparation of library, these tags were sequenced on MiSeq platform (Illumina, Inc., CA, USA) for paired-end reads of 250bp. DNA extraction, library construction and sequencing were conducted at Realbio Genomics Institute (Shanghai, China).

16s rRNA sequencing data analysis

Tags, trimmed of barcodes and primers, were further checked on their rest lengths and average base quality. 16S tags were restricted between 220 bp and 500 bp such that the average Phred score of bases was not lower than 20 (Q20) and no more than 3 ambiguous N. The copy number of tags was enumerated and redundancy of repeated tags was removed. Only the tags with frequency of more than 1 were clustered into OTUs, each of which had a representative tag. Operational Taxonomic Units (OTUs) were clustered with 97% similarity using UPARSE (17) and chimeric sequences were identified and removed using Usearch (version 7.0) (18). Taxonomic assignment was done by RDP Classifier (19) against the RDP database (http://rdp.cme.msu.edu/) using confidence threshold of 0.8. OTU profiling table and alpha/beta diversity analyses were also achieved by python scripts from Qiime (20). The α-diversity indices evaluating gut microbial community richness (the Chao1 index) and community diversity (the Shannon and Simpson indexes) were calculated from rarefied data. Principal coordinate analysis (PCoA) based on UniFrac distance and permutational multivariate analysis of variance (PERMANOVA) with 999 permutations were performed to compare the global microbiota composition between groups at genus level. Linear discriminant analysis effect size (LEfSe) (21), with default parameters and the logarithmic LDA score threshold of 2.0, was employed for differential abundance analysis at various taxonomic levels without pre-filtering based on abundances. Spearman’s rank-based correlations were used for assessing the correlations between microbial taxa and clinical metadata.

### Functional analysis of gut microbiota

PICRUSt (22) was used for predicting gut microbiota functional profiles as Kyoto Encyclopedia of Genes and Genomes (KEGG) Orthology and further collapse into KEGG pathways. LEfSe (21) was used to identify significantly enriched pathways (p<0.05, Kruskal-Wallis test) for the two TCM syndromes of MS and the healthy control group.

### Statistical analysis

Chi square test was used for comparisons of categorical data, Kruskal-Wallis test was used for comparing age (non-normally distributed) and one-way ANOVA followed by *post-hoc* Tukey’s test was used for comparing body mass index (normally distributed) using R package *stats*. Kruskal-Wallis test and Wilcoxon rank sum test were used to compare microbial alpha diversity indexes of the metabolic syndrome groups and the control group, as well as between the two metabolic syndrome groups. LEfSe or Wilcoxon rank sum test of two tailed distribution was used to test for significantly different taxa between the metabolic syndrome groups and the control group, as well as between the two metabolic syndrome groups. Multiple hypothesis testing was corrected using false discovery rate (FDR) method (23), for Wilcoxon rank sum test to screen significant genera and for Spearman correlation analyses between genera and clinical indicators. Raw p value of 0.05 was deemed significant unless otherwise stated.

## Results

### Basic characteristics of study participants

From March 2018 to May 2019, 60 MS patients who met the inclusion criteria were collected from the outpatient and inpatient MS patients at Department of Traditional Chinese Medicine, Shanghai Sixth People’s Hospital Affiliated to Shanghai Jiao Tong University School of Medicine. This included 29 cases of qi-yin deficiency syndrome (QYLX) and 31 cases of phlegm-dampness depression syndrome (TSZE). At the same time, 30 healthy participants were recruited, including 16 males and 14 females. All volunteers provided informed consent participating in the study. Clinical characteristics of the 90 human subjects are shown in **Table 1**. There was no significant difference in age or sex between the patients with MS and the control group (p>0.05). There was no significant difference in clinical indicators between the QYLX and the TSZE groups (p>0.05).

**Table 1:** Comparison of age, sex and clinical indicators between MS patients and control group.

Variable	QYLX (n=29)	TSZE (n=31)	Control group (n=30)	*P* value
**Gender**				
Male (%)	11 (37.9%)	13 (41.9%)	16 (53.3%)	
Female (%)	18 (62.1%)	18 (58.1%)	14 (46.7%)	
**Age (years)**	54.93 ± 9.651	54.30 ± 8.871	43.97 ± 18.31	0.069^1^
**BMI (kg/m^2^)**	26.33 ± 0.62	25.28 ± 0.55	22.02 ± 0.49	<0.001^2^
**Liver enzyme index**				
ALT (U/L)	26.61 ± 16.26	24.11 ± 13.95		0.539
AST (U/L)	22.43 ± 7.21	22.07 ± 7.35		0.855
**Renal function index**				
SCr (µmol/L)	63.27 ± 13.69	64.00 ± 14.80		0.849
BUN (mmol/L)	5.38 ± 1.33	5.87 ± 1.60		0.219
UA (µmol/L)	357.85 ± 86.04	353.96 ± 98.35		0.877
**Blood lipid index**				
TC (mmol/L)	4.98 ± 1.09	5.21 ± 1.11		0.433
TG (mmol/L)	1.93 ± 1.18	2.48 ± 1.69		0.163
HDL (mmol/L)	1.21 ± 0.32	1.16 ± 0.37		0.601
LDL (mmol/L)	3.01 ± 0.91	3.12 ± 1.10		0.685
**Glucose metabolism index**				
FPG (mmol/L)	5.89 ± 1.61	6.29 ± 1.67		0.361

^1^Data do not conform to normal distribution; Kruskal-Wallis test p=0.069.

^2^Data conform to normal distribution; one-way ANOVA p=9.7e-07 (post-hoc Tukey’s test: QYLX vs control: p=1.4e-06; TSZE vs control: p=1.9e-04; QYLX vs TSZE: p=0.378).

ALT, alanine aminotransferase; AST, aspartate aminotransferase; SCr, serum creatinine; BUN, blood urea nitrogen; UA, Uric Acid; TC, total cholesterol; TG, triglyceride; HDL, high density lipoprotein cholesterol; LDL, low density lipoprotein cholesterol; FPG, fasting plasma glucose. Student's t test was used for comparison of other data between the two MS groups.

### Comparison of gut microbiota structure among patients of 2 TCM subtypes of MS and the healthy control participants

**Figure 1:**
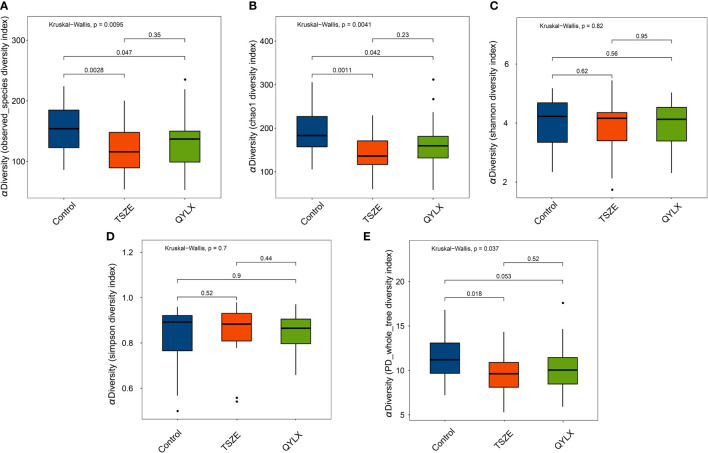
Comparison of microbiota alpha diversity among different TCM syndromes of MS and healthy control. Rarefied data are used to calculate different diversity indexes as **(A)** observed species; **(B)** Chao1 diversity; **(C)** Shannon diversity; **(D)** Simpson diversity; **(E)** phylogenetic diversity. Kruskal-Wallis test and Wilcoxon rank sum test were used for statistical comparison, and p value < 0.05 is considered statistical significance. Boxplots show median (centerlines), lower/upper quartiles (box limits), whiskers (the last data points 1.5 times interquartile range (IQR) from the lower or upper quartiles), and notches (95% confidence interval for the medians). QYLX, qi-yin deficiency syndrome; TSZE, phlegm-dampness syndrome.

We then performed 16S rRNA sequencing for the 90 participants to investigate the differences of gut microbiota among QYLX, TSZE patients and healthy control participants. This generated a total of 3242982 clean reads (mean 36033; s.d. 1822) after quality control. The Good’s coverage estimator for all samples was above 99.7%, indicating the sufficiency of sequencing depth to recover the vast majority of the microbiota community in all samples. The comparisons of microbiota alpha diversity indexes showed significant differences in chao1 index, observed species index and phylogenetic diversity (PD whole tree) between each MS group and the healthy control group (**Figure 1**) (p<0.05, Wilcoxon rank sum test), except for the comparison of phylogenetic diversity between QYLX and control (p=0.053). However, there were no significant differences in alpha diversity between the QYLX and the TSZE groups.

**Figure 2:**
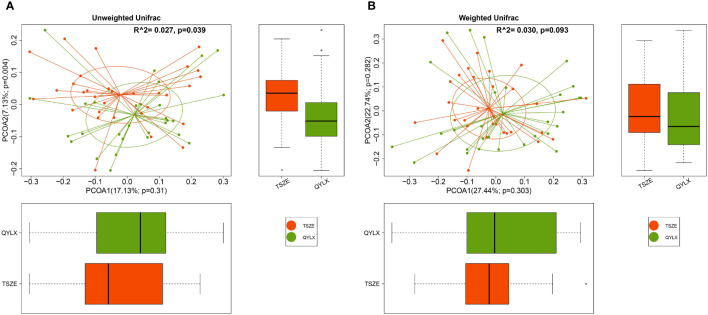
Principal coordinate analysis (PCoA) comparing microbiota beta diversity between the QYLX and TSZE groups. Genus-level abundance data are used for calculating both **(A)** unweighted and **(B)** weighted UniFrac distances. The horizontal and vertical box plots show the sample distribution on the first and second principal coordinates for each group, respectively. Statistical comparison of microbial communities and each principal coordinate between two groups were based on PERMANOVA and Wilcoxon rank sum test, respectively. P value < 0.05 is considered statistical significance. QYLX, qi-yin deficiency syndrome; TSZE, phlegm-dampness syndrome.

To further investigate the differences in the overall gut microbiota composition among different groups, we calculated and compared genus-level beta diversities using both unweighted and weighted UniFrac distances. Principal coordinate analysis (PCoA) and PERMANOVA showed small, yet statistically significant differences between each of the MS groups and the control group (**Supplementary Figure 1**) (QYLX vs Control: p=0.001, R^2^ = 0.051 for unweighted and p=0.032, R^2^ = 0.049 for weighted UniFrac; TSZE vs Control: p=0.001, R^2^ = 0.075 for unweighted and p=0.001, R^2^ = 0.090 for weighted UniFrac). It was also observed that the TSZE group was more different to the healthy control group than the QYLX group, using either unweighted or weighted UniFrac (**Supplementary Figure 1**). Notably, the two TCM syndromes of MS showed significant differences (p=0.039, R^2^ = 0.027, PERMANOVA) in intestinal microbiota structure when using unweighted UniFrac distance (**Figure 2A**), and specifically in the second principal coordinate of PCoA (p=0.004, Wilcoxon rank sum test). This association between microbiota diversity and different TCM subtypes of MS was not obvious with weighted UniFrac distance that considers microbial abundances (p=0.093, R^2^ = 0.030, PERMANOVAs) (**Figure 2B**). This finding implies that the TSZE and QYLX groups may share more similarity in the highly abundant taxa but are more distinct in the low abundant genera.

### Differentially abundant taxa among patients with distinct TCM subtypes of MS and healthy control participants

Next, we performed pairwise differential abundance analysis among TSZE, QYLX and healthy control groups, aiming to identify specific bacterial taxa associated with different TCM syndromes of MS. Compared to the control group, LEfSe analysis identified 25 (10 enriched and 15 depleted) and 43 (28 enriched and 15 depleted) significant taxa in patients with QYLX and TSZE, respectively (**Supplementary Figure 2, 3**, **Supplementary Table 1**). The higher number in comparing TSZE against the control group is consistent with the observation from ecological diversity comparison above that the TSZE group was more different to the healthy control group than the QYLX group was. Moreover, some taxa are commonly enriched (e.g. *Flavonifractor* and *Epsilonproteobacteria*) or depleted (e.g. *Paraprevotella* and *Faecalibacterium*) in both TCM syndromes compared to the healthy control group, implying the shared intestinal microbiota and pathophysiological characteristics of the two MS subtypes.

**Figure 3:**
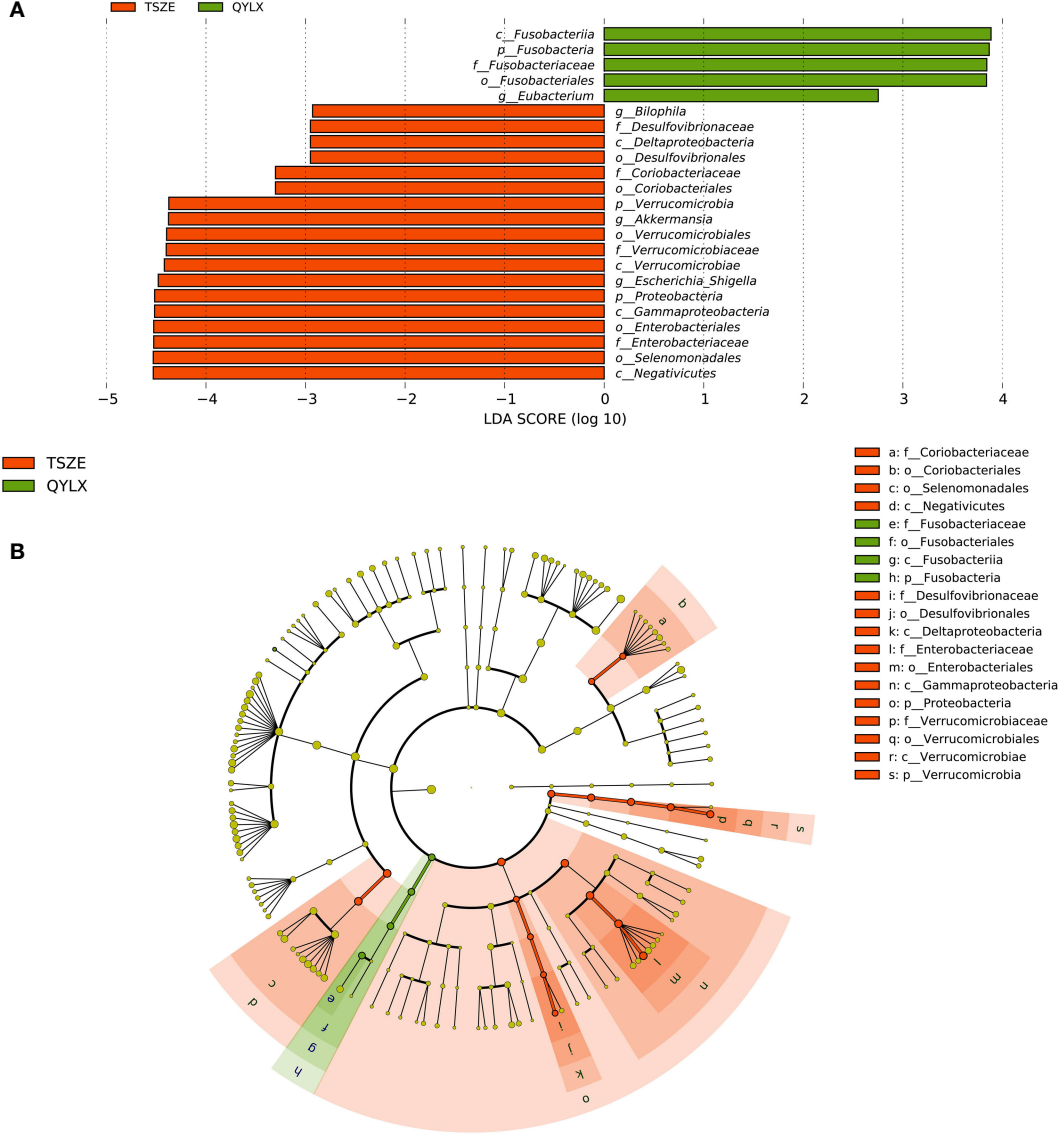
Specific microbiota taxa at various taxonomic ranks differentially enriched in QYLX and TSZE patients. **(A)** The full list of significant microbial taxa is identified with LefSe (linear discriminant analysis effect size), at p value < 0.05 and log10-transformed LDA score > 2. **(B)** A cladogram showing the enriched microbial taxa on a phylogenetic tree. The color indicates which branch of the phylogenetic tree more significantly represents a certain group. Green: microbial taxa enriched in QYLX group; orange: microbial taxa enriched in TSZE group. QYLX, qi-yin deficiency syndrome; TSZE, phlegm-dampness syndrome.

However, there are also microbial signatures unique to either QYLX or TSZE. Indeed, a direct comparison between the two TCM syndromes revealed a cluster of significantly differentially abundant taxa (5 QYLX-enriched and 18 TSZE-enriched), some of which are possibly related to the distinct clinical manifestations of the two TCM syndromes (**Supplementary Table 1**). According to **Figure 3**, the microbial taxa enriched in TSZE group with the highest LDA score was the class *Negativicutes* (p=0.012), followed by *Selenomonadales*, *Enterobacteriaceae*, *Enterobacteriales*, *Gammaproteobacteria*, *Proteobacteria*, etc. Conversely, the taxa enriched in QYLX group included the genus *Eubacterium*, the family *Fusobacteriaceae* and its affiliated higher taxonomic ranks.

In addition, we used Wilcoxon rank sum test to screen significantly differentially abundant genera between healthy and each TCM syndrome of MS, as well as between the two TCM syndromes. Similar conclusions were obtained regarding the differences in the intestinal microbiota mentioned above. Compared to the control group, this identified 7 and 11 significant genera after correcting for multiple hypothesis testing (FDR-correct p<0.05) in patients with QYLX and TSZE, respectively (**Supplementary Figure 4**, **Supplementary Table 1**). Even though patients of both TCM syndromes of MS differed considerably from healthy participants, *Akkermansia* was the only significant genus between them after FDR correction (FDR-corrected p<0.05).

### Gut microbiota genera correlated with MS-related clinical measurements differently in QYLX and TSZE

**Figure 4:**
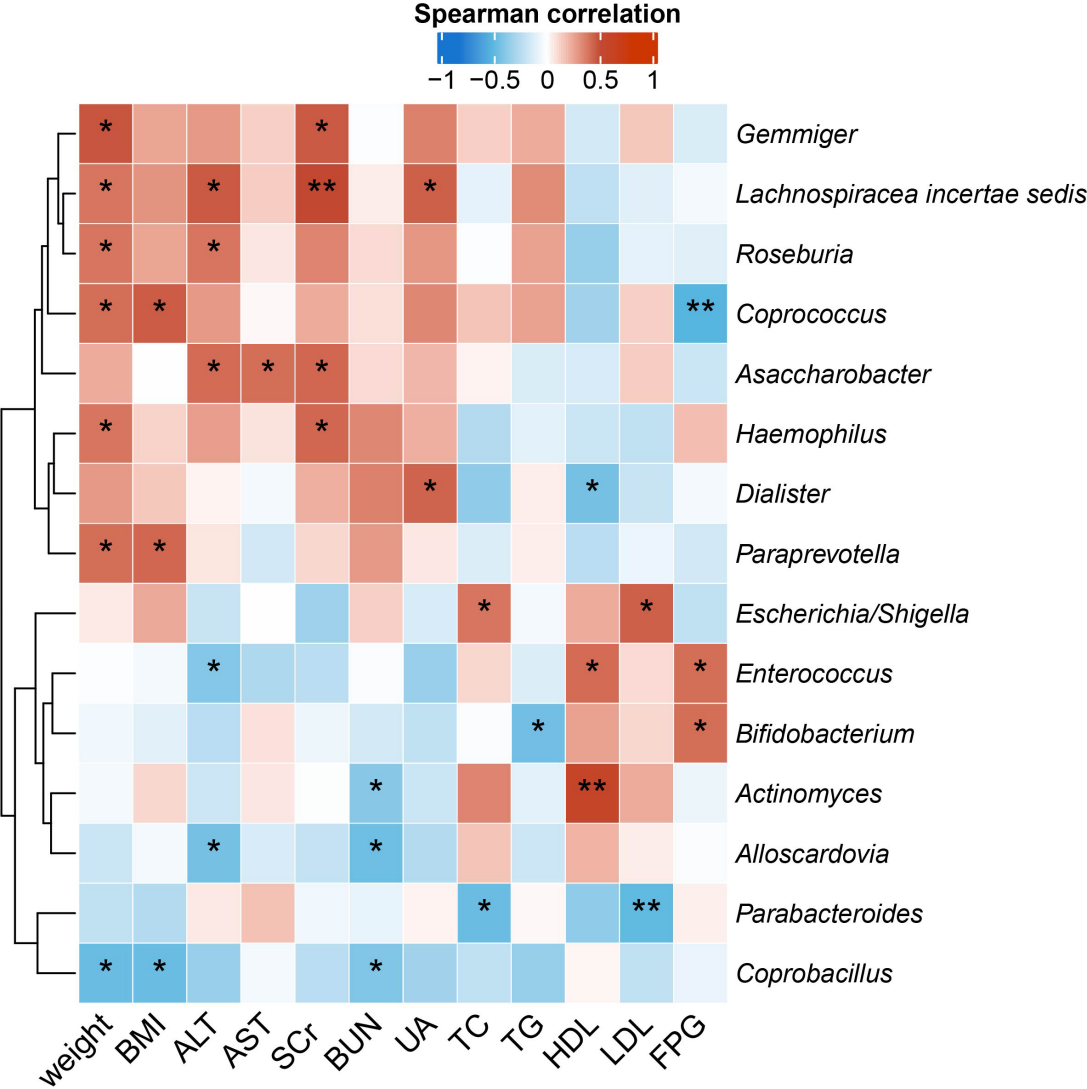
Correlations between specific genera and MS-related clinical measurements in the QYLX group. Spearman’s rank-based correlations are calculated using samples from the QYLX group between each genus and clinical metadata related to anthropometrics, liver and kidney functions, lipid metabolism, and glucose homeostasis. Only genera with at least two significant correlation (p < 0.05) are included. Red, positive correlations; blue, negative correlations. *p< 0.05; **p< 0.01. ALT, alanine aminotransferase; AST, aspartate aminotransferase; SCr, serum creatinine; BUN, blood urea nitrogen; UA, Uric Acid; TC, total cholesterol; TG, triglyceride; HDL, high density lipoprotein cholesterol; LDL, low density lipoprotein cholesterol; FPG, fasting plasma glucose.

**Figure 5:**
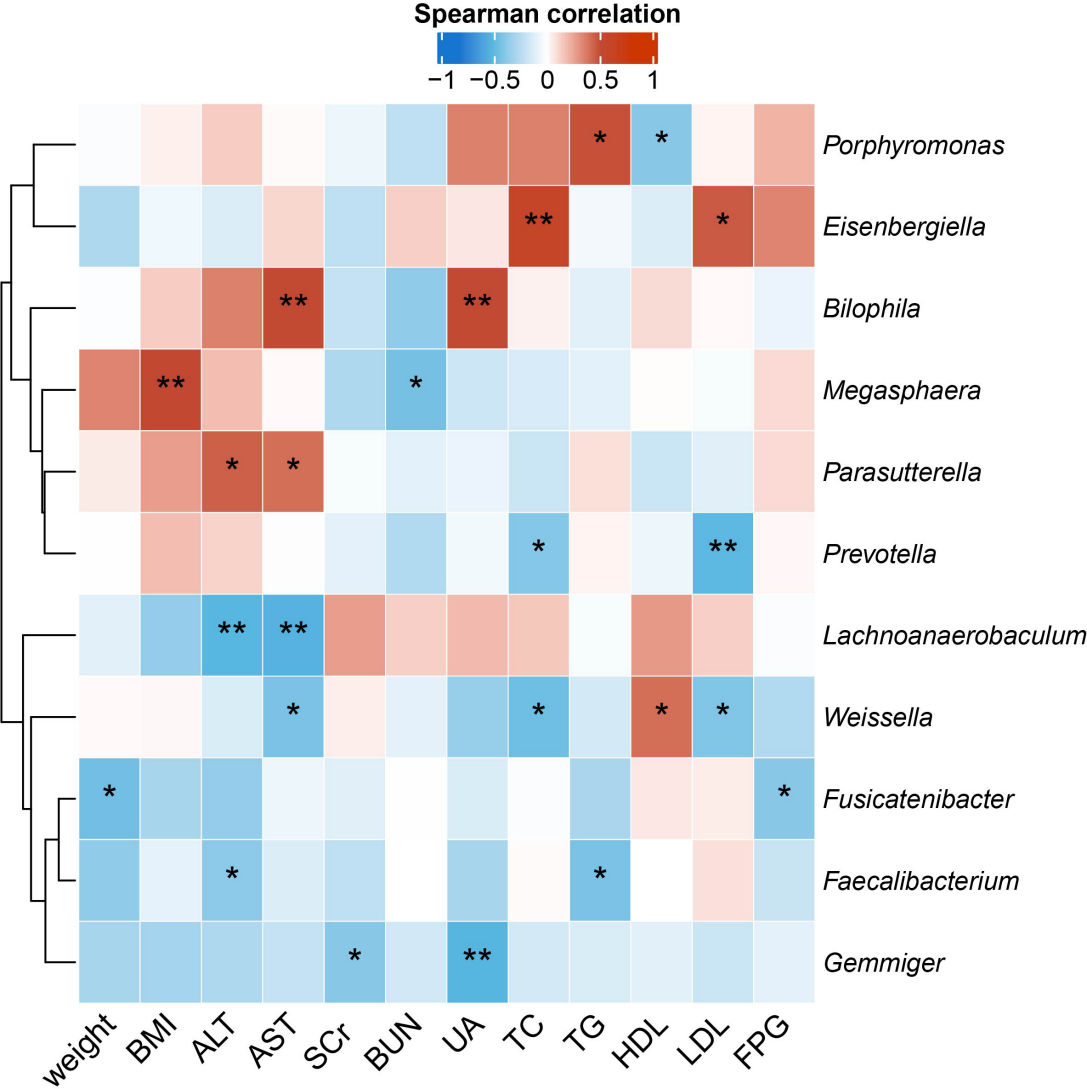
Correlations between specific genera and MS-related clinical measurements in the TSZE group. Spearman’s rank-based correlations are calculated using samples from the TSZE group between each genus and clinical metadata related to anthropometrics, liver and kidney functions, lipid metabolism, and glucose homeostasis. Only genera with at least two significant correlation (p < 0.05) are included. Red, positive correlations; blue, negative correlations. *p< 0.05; **p< 0.01. ALT, alanine aminotransferase; AST, aspartate aminotransferase; SCr, serum creatinine; BUN, blood urea nitrogen; UA, Uric Acid; TC, total cholesterol; TG, triglyceride; HDL, high density lipoprotein cholesterol; LDL, low density lipoprotein cholesterol; FPG, fasting plasma glucose.

To identify the microbial taxa that are potentially associated with disease phenotypes and pathophysiology, we performed spearman correlation analysis between intestinal microbial genera and a panel of clinical metadata related to anthropometrics, liver and kidney functions, lipid metabolism, and glucose homeostasis. For the QYLX group, we observed 36 significant correlations from 15 genera; weight, alanine transaminase (ALT), and serum creatinine (SCr) had the top 3 highest number of related genera, while *Lachnospiracea incertae sedis*, *Coprococcus*, *Asaccharobacter*, *Enterococcus*, and *Coprobacillus* had the most correlations with clinical data (**Figure 4**). In comparison, the TSZE group had fewer correlations (n=24) from fewer genera (n=11); aspartate transaminase (AST), ALT, total cholesterol (TC), and low-density lipoprotein (LDL) were correlated with the highest number of genera, while *Weissella* had most correlations with clinical measures that were mainly related to lipid metabolism (**Figure 5**). Notably, almost no correlations were commonly found between the QYLX and the TSZE groups, except the correlation between *Gemmiger* and SCr but with opposite direction. The highly distinct correlation patterns remained when we re-performed the correlation analysis using microbial abundances after centered log-ratio transformation that deals with data compositionality. Within the bacteria correlated to clinical profile, two genera (*Coprococcus* and *Haemophilus*) were also significantly differentially abundant between the QYLX group and healthy control (**Supplementary Figure 4**), while three genera (*Faecalibacterium*, *Gemmiger*, and *Prevotella*) were observed for the TSZE comparison. In summary, the QYLX and the TSZE groups differ in gut microbiota composition and microbial correlations with clinical measures related to MS, implying the association between gut microbiota and TCM manifestations of metabolic syndrome.

### Differential functions between TSZE and QYLX

**Figure 6:**
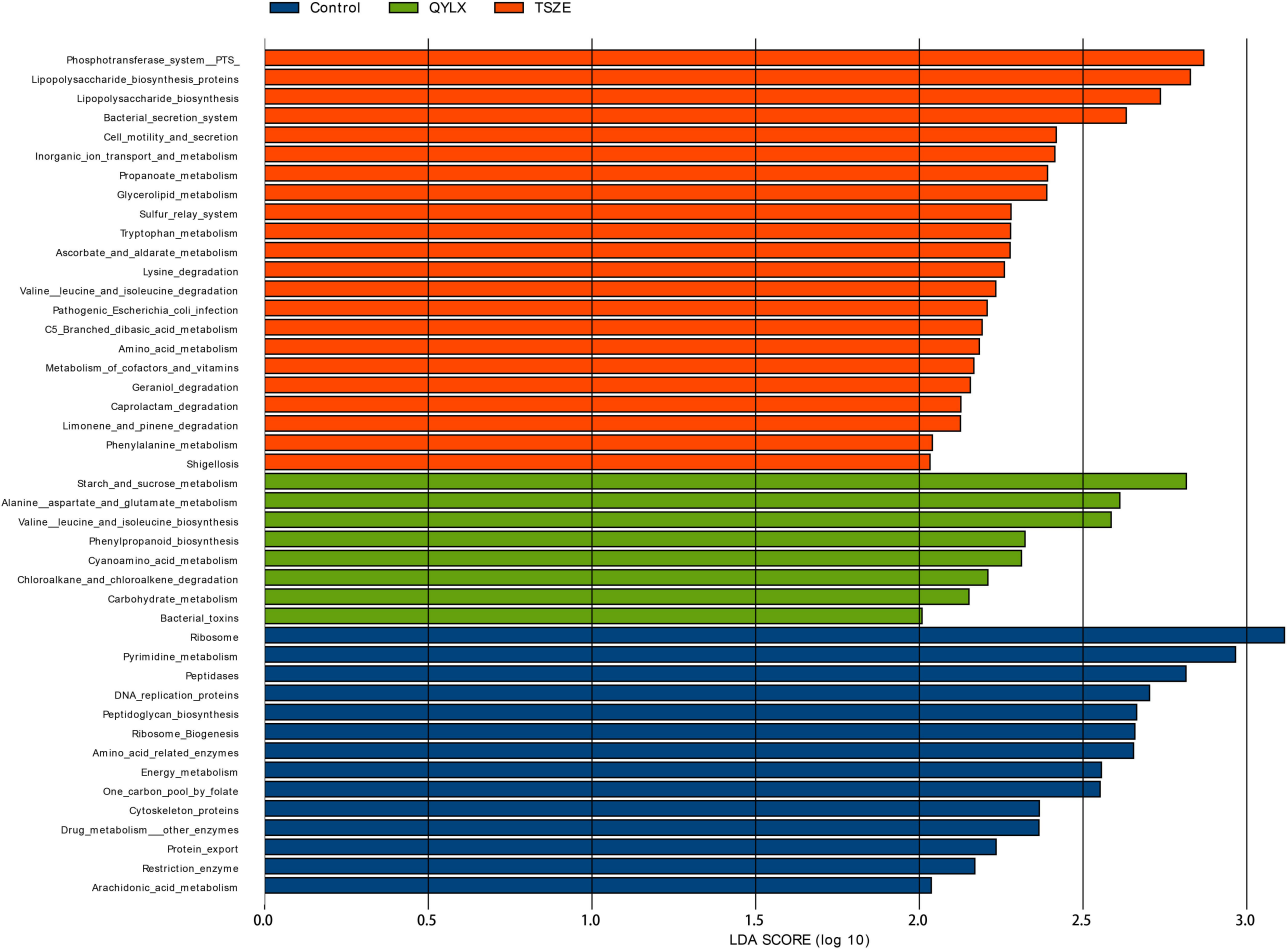
Specific microbiota functions differentially abundant among QYLX, TSZE patients and healthy control. The full list of significant KEGG pathways enriched in each of the 3 groups is identified with LefSe (linear discriminant analysis effect size), at p value < 0.05 and log10-transformed LDA score > 2. Green: microbial taxa enriched in QYLX group; orange: microbial taxa enriched in TSZE group; blue: microbial taxa enriched in healthy control, thus depleted in both TCM syndromes of MS. QYLX, qi-yin deficiency syndrome; TSZE, phlegm-dampness syndrome.

Having compared the microbiota structure and compositions, we then ought to investigate the microbiota functional differences among the TSZE, QYLX and healthy control groups. The KEGG pathways were predicted with PICRUSt, followed by LEfSe to identify signature pathways of each group (**Figure 6**). In patients with QYLX, gut microbiota was enriched in functions related to carbohydrate metabolism (starch and sucrose metabolism; carbohydrate metabolism). In the TSZE group, microbiota functions related to lipopolysaccharide (LPS) synthesis (such as lipopolysaccharide biosynthetic protein and lipopolysaccharide biosynthesis) were more abundant, and the gut microbiota-derived LPS has been implicated in inflammation and obesity (24). The metabolism of cofactors and vitamins was also enriched in the TSZE group.

Moreover, the QYLX and TSZE groups showed large differences in amino acid metabolism of gut microbiota: alanine, aspartate and glutamate metabolism, as well as valine, leucine and isoleucine biosynthesis pathway were more abundant in QYLX group; while in TSZE patients, tryptophan metabolism, lysine degradation, valine leucine and isoleucine degradation, and phenylalanine metabolism were enriched. Such discrepancy in the amino acid metabolism of intestinal microbiota is possibly related to the different TCM syndromes of MS and their clinical manifestations.

## Discussion

In this study, we performed a cross-sectional analysis of human gut microbiota in patients with two different TCM syndromes (QYLX and TSZE) of MS, and compared their differences with healthy participants. Through high throughput 16S rRNA sequencing of fecal samples from 60 MS patients and 30 healthy control participants, it was found that the QYLX and TSZE groups differ from the healthy control group in the overall gut microbiota composition, and in specific microbial taxa and functional pathways. The TSZE group seemed to have higher dissimilarity from the healthy control group than the QYLX group regarding the microbiota composition and functionality. Moreover, significantly differentially abundant taxa and distinct BMI-correlated taxa were observed between QYLX and TSZE groups in our exploratory study. These findings, though remain to be confirmed by future studies on a larger scale, may suggest the potential contribution of gut microbiota to the distinction between the two TCM syndromes.

Despite numerous efforts to understand and treat MS, its exact pathogenesis is not clear yet (25). Previous studies have demonstrated the important regulatory role of intestinal microbiota in the pathogenesis of MS (26, 27).Specific changes in microbial taxa have been associated with MS, e.g. the increase of *Lactobacillus* and decrease of *Faecalibacterium* in patients with MS (26, 28), which were also observed in our QYLX and TSZE syndromes of MS, respectively. The relative abundance of *Akkermansia* was reported to be lower in MS patients (26, 29), but we observed here a significant increase in the patients with TSZE syndrome of MS compared to the healthy control participants. The change of gut microbiota can affect the host’s metabolism through several routes such as gut barrier integrity, production of metabolites affecting satiety and insulin resistance, epigenetic factors, and bile acid metabolism and subsequent changes in metabolic signaling (30). Current research into different components of MS and gut microbiota is getting more and deeper, and our understanding of the pathophysiological mechanism of MS is constantly being updated. Central obesity is one of the main clinical manifestations of MS. Le Roy et al. (31) found seven bacterial genera related to visceral fat mass, of which *Coprococcus* and *Ruminococcus* showed significant negative correlations. Interestingly, *Coprococcus* and *Ruminococcus* were reduced here in patients with both QYLX (though not statistically significant for *Ruminococcus*) and TSZE syndromes of MS (**Supplementary Figure 4**), and were also reported to be lower in elderly patients with MS (28). A recent metagenome-wide association study has demonstrated the strong connection between visceral fat, a hallmark of central obesity, with gut microbiota composition and functionality (32). Specifically, they found that the microbiota biosynthesis of phenylalanine and LPS are positively correlated with visceral fat area, waist and waist-to-hip ratio. In our study, both pathways were increased in the patients with TSZE syndrome of MS (**Figure 6**). The changes in microbiota metabolites or microbial components might be also responsible for the impaired intestinal mucosal barrier function and increased intestinal permeability in obese people (33–35). Microbial metabolism of tryptophan, which was shown to regulate gut barrier function *via* the aryl hydrocarbon receptor (36), was enriched in the TSZE syndrome of MS.

Diabetes, hyperlipidemia and hypertension are important parts of MS and their relationship with intestinal microbiota has been widely studied. Compared with non-diabetic patients, adult patients with type II diabetes patients have an imbalanced intestinal microecology and decreased abundance of butyrate-producing bacteria in the intestine (37, 38). Interestingly, the aforementioned genera *Coprococcus* and *Ruminococcus*, which were decreased in patients with the TCM syndromes of MS, are also butyrate-producing bacteria (34). Amino acids and their byproducts were suggested to play a key role in obesity and its complications including insulin resistance, hyperglycemia, and dyslipidemia (39). Here we found distinct pathways of amino acid metabolism to be enriched in patients with different TCM syndromes of MS (**Figure 6**). Through shotgun metagenomic analysis of a cohort of 196 Chinese individuals, Li et al. found decreased microbial richness and diversity, altered microbiota composition and functionality (increase in LPS biosynthesis and export, phenylalanine biosynthesis, phosphotransferase system, and secretion system, among others), in both pre-hypertensive and hypertensive populations (40). They further transplanted fecal microbiota from hypertensive human donors to germ-free mice and observed elevated blood pressure, demonstrating the causal role of intestinal microbiota in the pathogenesis of hypertension. In our study, we also found decreased microbiota richness in patients with the two TCM syndromes of MS compared to healthy participants (**Figure 1**), although no significant differences were observed in Shannon or Simpson diversity. Microbial functions including LPS biosynthesis, phenylalanine metabolism, secretion system, and phosphotransferase system were enriched as well in patients with the TSZE syndrome of MS.

This pilot study demonstrates again the vital role of gut microbiota in metabolic syndrome, highlights its contribution to the differentiation between two TCM syndromes of MS, and offers functional insights into such differentiation based on inferred functions such as amino acid metabolism and LPS biosynthesis. Similar studies of gut microbiota have been performed in other TCM syndromes such as spleen-yang-deficiency syndrome (41), kidney-yang-deficiency syndrome (42), as well as different TCM syndromes of ulcerative colitis (13) and colorectal cancer (14). Future research may investigate the differences in microbiota metabolism of amino acids and the serum or stool amino acid levels in MS patients with different TCM syndromes, by employing shotgun metagenomics approach coupled with targeted or untargeted metabolomics analysis. The exploration of mechanistic differences can shed light on the personalized management of patients with metabolic syndrome and future intervention studies using TCM, especially for microbiota-targeted therapies. However, this study has also limitations. The lack of detailed information on medication usages such as antihypertensive drugs and hypoglycemic drugs is a major limitation. A longer absence period of antibiotics usage can be used in future studies to reduce its confounding effect. Future work may use shotgun metagenomic sequencing, together with a metabolomics, for deeper and more accurate characterization of gut microbiota composition and functionality or functional readout. Given the limited sample size, larger cohorts with multi-center design are needed in the future for validation of our findings here.

## Data availability statement

The datasets presented in this study can be found in online repositories. The names of the repository/repositories and accession number(s) can be found below: https://www.ncbi.nlm.nih.gov/, PRJNA809501.

## Ethics statement

The clinical study was reviewed and approved by the ethics committee of Shanghai Sixth People’s hospital. The patients/participants provided their written informed consent to participate in this study.

## Author contributions

BW, YN, and ZY designed and supervised the study. HS, LuZ, JR, QZ, KL, and LiZ finished data collection and analysis. TX, YN and BW provided technical guidance for the whole work. YN and HS. wrote the manuscript. All authors contributed to the study and approved the final version of the manuscript.

## Funding

This work was supported by Shanghai High-level Talents Leading Plan of Traditional Chinese Medicine, Three-year Action Plan (2021-2023) of Shanghai Municipality for Further Accelerating the Inheritance, Innovation and Development of Traditional Chinese Medicine [grant number: ZY(2021-2023)-0205-04], Construction of East China Area and Municipal TCM Specialist Disease Alliance [grant number: ZY(2021-2023)-0302] to BW.

## Conflict of interest

The authors declare that the research was conducted in the absence of any commercial or financial relationships that could be construed as a potential conflict of interest.

## Publisher’s note

All claims expressed in this article are solely those of the authors and do not necessarily represent those of their affiliated organizations, or those of the publisher, the editors and the reviewers. Any product that may be evaluated in this article, or claim that may be made by its manufacturer, is not guaranteed or endorsed by the publisher.
