# Supplementary material for: Study on the differences of gut microbiota composition between phlegm-dampness syndrome and qi-yin deficiency syndrome in patients with metabolic syndrome
Source: Front Endocrinol (Lausanne). 2022 Nov 9;13:1063579. doi: 10.3389/fendo.2022.1063579 (PMC9682026; doi:10.3389/fendo.2022.1063579)
Supplement: Supplementary file 1 [file DataSheet_1.zip › Supplementary Figures and Tables/Supplementary figures.pdf]

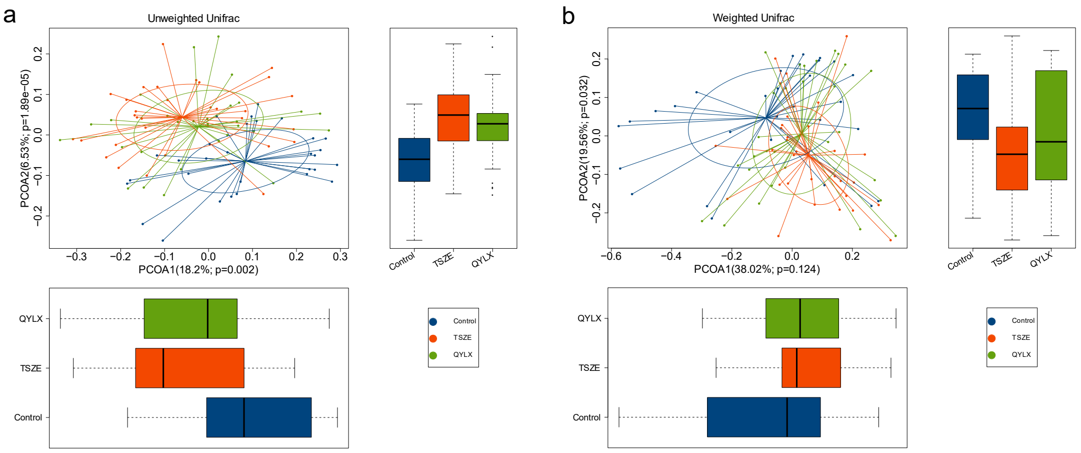

Supplementary Figure 1. Principal coordinate analysis (PCoA) comparing microbiota beta diversity among QYLX, TSZE patients and healthy control. Genus-level abundance data are used for calculating both (**a**) unweighted and (**b**) weighted UniFrac distances. The horizontal and vertical box plots show the sample distribution on the first and second principal coordinate for each group, respectively. Statistical comparison of microbial communities and of each principal coordinate between two groups were based on PERMANOVA and Wilcoxon rank sum test, respectively. P value < 0.05 is considered statistical significance. QYLX, qi-yin deficiency syndrome; TSZE, phlegm-dampness syndrome.

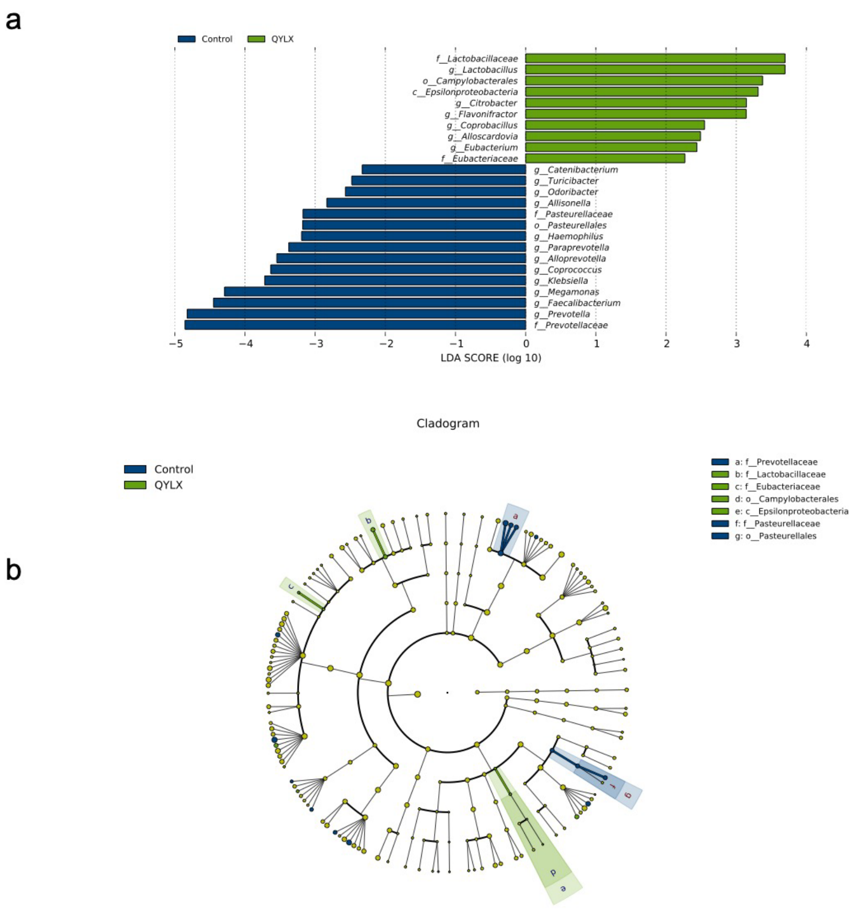

Supplementary Figure 2. Specific microbiota taxa at various taxonomic ranks enriched and depleted in QYLX patients. (**a**) The full list of significant microbial taxa is identified with LefSe (linear discriminant analysis effect size), at p value < 0.05 and log10-transformed LDA score > 2. (**b**) A cladogram showing the enriched microbial taxa on a phylogenetic tree. The color indicates which branch of the phylogenetic tree more significantly represents a certain group. Green: microbial taxa enriched in QYLX group; blue: microbial taxa enriched in healthy control, thus depleted in QYLX group. QYLX, qi-yin deficiency syndrome.

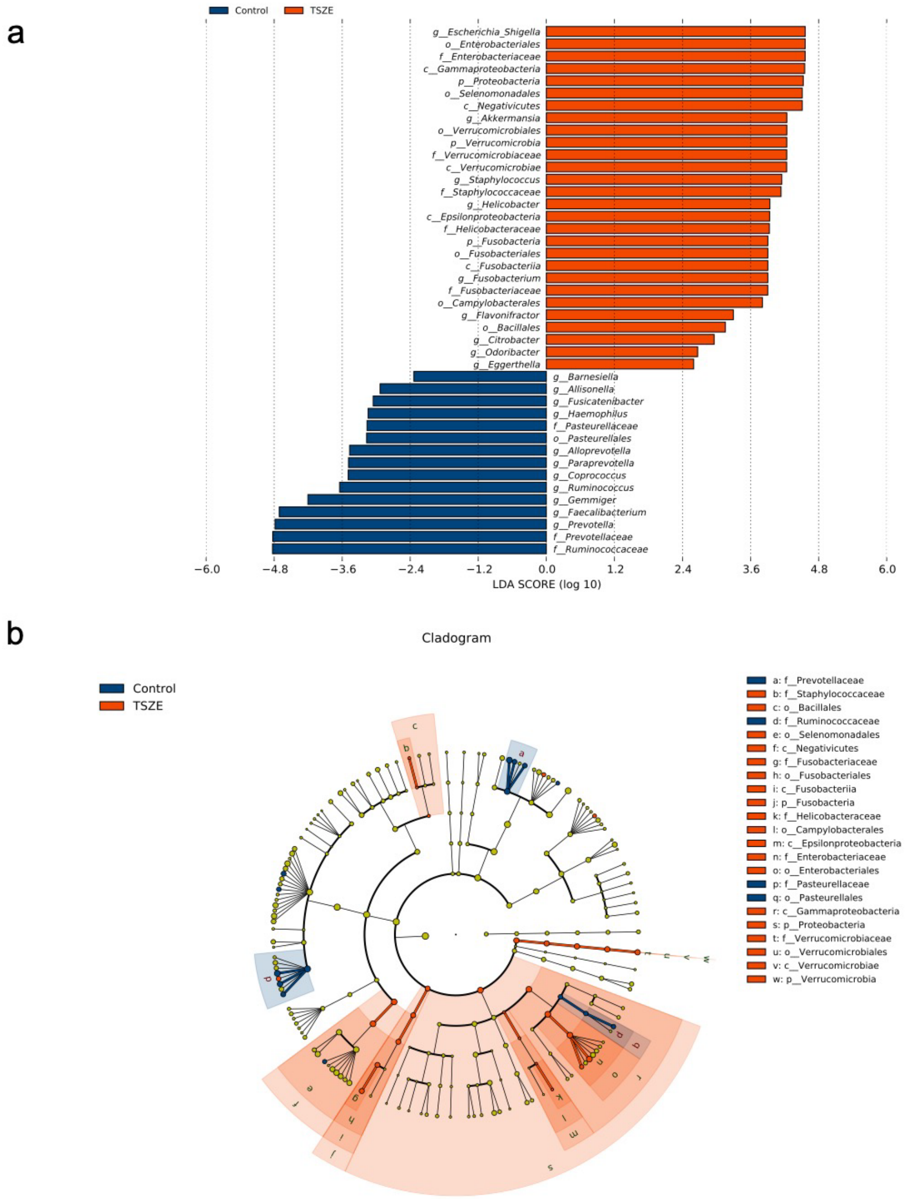

Supplementary Figure 3. Specific microbiota taxa at various taxonomic ranks enriched and depleted in TSZE patients. (**a**) The full list of significant microbial taxa is identified with LefSe (linear discriminant analysis effect size), at p value < 0.05 and log10-transformed LDA score > 2. (**b**) A cladogram showing the enriched microbial taxa on a phylogenetic tree. The color indicates which branch of the phylogenetic tree more significantly represents a certain group. Green: microbial taxa enriched in TSZE group; blue: microbial taxa enriched in healthy control, thus depleted in TSZE group. TSZE, phlegm-dampness syndrome.

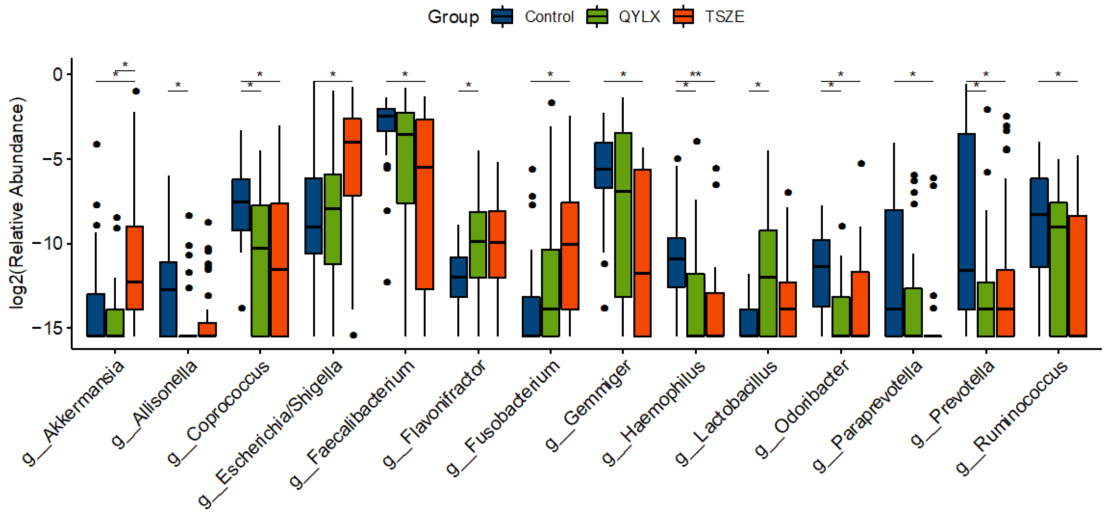

Supplementary Figure 4. Significantly differentially abundant genera among QYLX, TSZE patients and healthy control. Wilcoxon rank sum test was used to identity significantly differentially abundant genera (FDR-corrected $p < 0.05$) between QYLX group and healthy control, between TSZE group and healthy control, as well as between the QYLX and TSZE groups. Only the genera significant in at least one comparison and whose average abundances are above zero in both groups being compared are shown. Boxplots show median (centerlines), lower/upper quartiles (box limits), whiskers (the last data points 1.5 times interquartile range (IQR) from the lower or upper quartiles), and notches (95% confidence interval for the medians). *, FDR-corrected $p< 0.05$; **, FDR-corrected $p< 0.01$. QYLX, qi-yin deficiency syndrome; TSZE, phlegm-dampness syndrome.
